# Hotspot Analysis of Spatial Environmental Pollutants Using Kernel Density Estimation and Geostatistical Techniques

**DOI:** 10.3390/ijerph8010075

**Published:** 2010-12-30

**Authors:** Yu-Pin Lin, Hone-Jay Chu, Chen-Fa Wu, Tsun-Kuo Chang, Chiu-Yang Chen

**Affiliations:** 1 Department of Bioenvironmental Systems Engineering, National Taiwan University, 1, Section 4, Roosevelt Road, Da-an District, Taipei City 106, Taiwan; E-Mails: yplin@ntu.edu.tw (Y.-P.L.); tknchang@ntu.edu.tw (T.-K.C.); 2 Department of Horticulture, National Chung Hsing University, 250, Kuo Kuang Road, Taichung 402, Taiwan; E-Mail: cfwu@dragon.nchu.edu.tw (C.-F.W.); 3 Department of Safety, Health and Environmental Engineering, Ming Chi University of Technology, 84, Gungjuan Road, Taishan, Taipei 24301, Taiwan; E-Mail: chiuyang@mail.mcut.edu.tw (C.-Y.C.)

**Keywords:** kernel density estimation (KDE), indicator Kriging (IK), sequential indicator simulation (SIS), heavy metal, soil contaminant

## Abstract

Concentrations of four heavy metals (Cr, Cu, Ni, and Zn) were measured at 1,082 sampling sites in Changhua county of central Taiwan. A hazard zone is defined in the study as a place where the content of each heavy metal exceeds the corresponding control standard. This study examines the use of spatial analysis for identifying multiple soil pollution hotspots in the study area. In a preliminary investigation, kernel density estimation (KDE) was a technique used for hotspot analysis of soil pollution from a set of observed occurrences of hazards. In addition, the study estimates the hazardous probability of each heavy metal using geostatistical techniques such as the sequential indicator simulation (SIS) and indicator kriging (IK). Results show that there are multiple hotspots for these four heavy metals and they are strongly correlated to the locations of industrial plants and irrigation systems in the study area. Moreover, the pollution hotspots detected using the KDE are the almost same to those estimated using IK or SIS. Soil pollution hotspots and polluted sampling densities are clearly defined using the KDE approach based on contaminated point data. Furthermore, the risk of hazards is explored by these techniques such as KDE and geostatistical approaches and the hotspot areas are captured without requiring exhaustive sampling anywhere.

## 1. Introduction

Unfortunately, as a result of industrial activities, improper disposal of wastes, pollution of agricultural soils with heavy metals has become an increasingly serious problem throughout the world [1–4]. To understand the contamination risk, monitoring is a necessary and prohibitively costly process. Risk assessment at unsampled locations is of significant importance for the delineation of contamination areas [5–9]. However, the accuracy of risk estimation depends on the methodology used and various related factors.

Geostatistical analysis considers the concentration of a potentially hazard in soil as a regionalized variable in space. Geostatistics was developed as a means to describe spatial patterns of soil pollution by semivariograms and to predict the values of soil attributes at unsampled locations [10]. Geostatistical models could be used to estimate the spatial patterns of soil contaminant without measuring soil data in an entire area. The degree of contamination and hotspot areas for soils may vary with the methods used. For delineating hazardous areas, indicator kriging (IK) determines the spatial probability distribution of soil pollution in fields [6,11–16]. IK provides a non-parametric distribution estimated at an unsampled location directly using fixed thresholds and qualifies the spatial patterns of a hazardous risk. Moreover, stochastic simulation methods such as sequential indicator simulation (SIS), have been recently proposed to overcome the inherent limitations of IK [17–19]. The stochastic simulation method is based on a probabilistic model, and does not require any assumption for the shape of the conditional distribution and the systematically adds a stochastic noise component into the kriging model. Simulation with multiple realizations offers significant improvements over kriging techniques at sites with high data variations.

Hotspot mapping is used to help identify where soil pollution exists and comes from. Recently, Kernel density estimation (KDE) is one of the methods for analyzing the first order properties of a point event distribution [20–22], in part because it is easy to understand and implement. KDE has been widely used for hotspot analysis and detection. The objective of KDE is to produce a smooth density surface of point events over space by computing event intensity as density estimation [22–24]. Moreover, Schnabel and Tietje [23] applied the KDE method to spatially distributed heavy metal soil data and compared it with ordinary kriging. The results represent the interdependence between various heavy metal concentrations and additional site characteristics. Furthermore, the method could be a valuable supplement for the geostatistical uncertainty assessments.

The purpose of this study was to propose alternative approaches in searching for pollutant hotspots. The primary objective of the present work was to investigate proposals for delineating soil pollutant hazards. First, KDE identifys the hotspots of soil pollutions based on the hazardous metal sampling data. Then, IK and SIS generate a hazard probability map based on the samples for management. A study case from a field survey is provided to estimate the probability maps for hazard delineation. It is expected that results can give references for identification of hazardous areas.

## 2. Methods and Materials

Kernel density estimation is used to identify the location, spatial extent and intensity of soil pollution hotspots. Moreover, the spatial patterns of hazardous probability for heavy metals are estimated using geostatistical methods. The three methods are used for visualization of hotspots of soil pollutions in the case study. Study area and sampling of heavy metals will be discussed in the following sections.

### 2.1. Study Area and Soil Sampling

**Figure 1:**
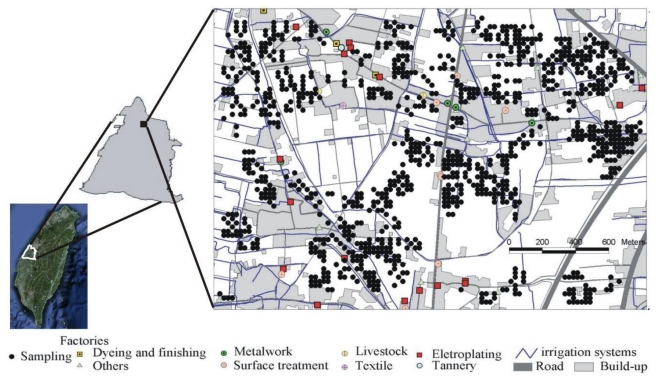
The study area and sampling sites.

The study area is in Changhua County, which is a critical agricultural region in Taiwan. Changhua city is in the east area and Lugang town lies to the west. Approximately 106 industrial plants are clustered in study area. Most industrial plants in the study area involve metalwork, electroplating, textile and metal surface treatment industries (Figure 1). The industrial plants have been suspected of discharging wastewater into irrigation channels in this study area [8,12,25]. The data of 1,309 topsoil (0–15 cm) samples containing concentrations of Cr, Cu, Ni, and Zn were obtained by the soil heavy metal investigation project carried by Taiwan’s Environmental Protection Administration (EPA), between February and August 2002. The sampling sites are shown in Figure 1.

Approximately 1 kg of soil was collected for each sample using a stainless steel spade and a plastic scoop and then stored in a plastic food bag. After air drying at room temperature, 3 g of each soil sample were disaggregated, sieved to 0.85 mm and ground to a fine 0.15 mm powder. Each 3 g milled sample was then digested for 2 h at room temperature with 7 mL HNO_3_ and 21 mL HCl (aqua regia, 1:3) to slowly oxidize organic matter in the soil. Next, the digest was filtered and made up to 100 mL with distilled water [15,16]. The levels of heavy metals in the samples were determined by Inductively Coupled Plasma-Optical Emission Spectrometers (ICP-OES).

### 2.2. Kernel Density Estimation (KDE)

The general form of a kernel density estimator in a 2-D space, termed KDE in the rest of this paper, is given by [22]:

(1) $$\lambda (s)=\sum_{i=1}^{n}\frac{1}{\pi r^{2}}k(\frac{d_{is}}{r})$$

where *λ* (*s*) is the density at location s, *r* is the search radius (bandwidth) of the KDE, *n* is the number of sampling points, *k* is the weight of a point *i* at distance *d_is_* to location *s. k* is usually modeled as a kernel function of the ratio between *d_is_* and *r.* In this study, we used a kernel with a Gaussian function given by:

(2) $$k(\frac{d_{is}}{r})=\{\begin{array}{cc} \frac{1}{\sqrt{2\pi }}\text{exp}(-\frac{{d_{is}}^{2}}{2r^{2}}), & if\;\;\;0<d_{is}\leq r\\ 0, & otherwise \end{array}$$

To identify the soil pollution hotspots, the KDE package based on ArcGIS software was used in the study.

### 2.3. Indicator Kriging (IK)

The IK estimates the probability that the concentration of a pollutant exceeds a specific control value at a given location [8,17]. The data (*z*(s)) are transformed into an indicator as follows:

(3) $$I(s,z_{c})=\{\begin{array}{cc} 1, & if\;\;\;z(s)\leq z_{c}\\ 0, & otherwise \end{array}$$

If the concentration of heavy metal [ *z*(*s*) ] exceeds *z_c_* then the indicator is 0, otherwise it is 1 [11]. The expected value of *I* (*s; z_c_* | (*n*)), conditional on *n* surrounding data, can be expressed as:

(4) $$E[I(s;z_{c}|(n))]=prob[z(s)\leq z_{c}|(n)]$$

The hazardous probability that exceeds *z_c_* can be expressed as:

(5) $$prob[z(s)>z_{c}|(n)]=1-prob[z(s)\leq z_{c}|(n)]$$

This ordinary indicator kriging estimator is:

(6) $$prob[z(s_{0})\leq z_{c}|(n)]=\sum_{\alpha =1}^{n}\lambda_{\alpha }I(s_{\alpha };z_{c})$$

where *I* (*s_α_*;*z_c_*) represents the indicator values at *x_α_* *; α* = 1,···,*n ; λ_α_* is the kriging weight of (*I* (*s_α_*;*z_c_*)) determined by solving the following kriging system:

(7) $$\sum_{\beta =1}^{n}\lambda_{\beta }\gamma_{i}(s_{\alpha }-s_{\beta };z_{c})+\mu =\gamma_{i}(s_{\alpha }-s_{0};z_{c})$$

(8) $$\sum_{\beta =1}^{n}\lambda_{\beta }=1$$

where *μ* is the Lagrange multiplier; *γ_i_* (*s_α_* − ;*s_β_*; *z_c_*) is the indicator variogram between indicator variables at the *α^th^* and *β^th^* sampling points; *γ_i_* (*s_α_* − ;*s*_0_; *z_c_*) is the variogram between the indicator variables, *i.e.*, the *α^th^* sampling point and *s*_0_; *α* =1,···,*n*.

### 2.4. Sequential Indicator Simulation (SIS)

In sequential indicator simulation, modeling of the *N*-point conditional cumulative distribution function (ccdf) is a sequence of *N* univariate ccdfs at each grid cell along a random path [25,26]. The SIS requires the following steps [17,25,26]:

1. Define a random path that visits each location of the domain once, in which all nodes {*s_i_*, *i* = 1,*N*} ··· discretizing the interest domain. A random visiting sequence ensures that no spatial continuity artifact is introduced into the simulation by a specific path visiting *N* nodes.
2. At the first visited nodes (*s*_1_):
  - Model, using either a parametric or nonparametric approach, the local ccdf of *z*(*s*_1_) conditional on *n* original data {*z*(*s_α_*),*α* =1,···,*n*}:
    (9) $$F_{Z}(s_{1};z_{1}|(n))=prob\{z(s_{1})\leq z_{1}|(n)\}$$
  - Generate, via the Monte Carlo drawing relation, a simulated value *z*^(^*^l^*^)^ (*s*_1_) from this ccdf *F_Z_* (*s*_1_ ; *z*_1_ | (*n*)), and add it to the conditioning data set, now of dimension *n* +1, to be used for all subsequent local ccdf determinations.
3. At the *i*^th^ node *s_i_* along the random path:
  - Model the local ccdf of *z*(*s_i_*) conditional on *n* original data and the *i −*1 near previously simulated values:
    (10) $$\left\{z^{\left(l\right)}(s_{j}),j=1,\cdots ,i-1\}:F_{Z}(s_{i};z_{i}|(n+i-1))=prob\{z(s_{i})\leq z_{i}|(n+i-1)\right\}$$
  - Generate a simulated value *z*^(^*^l^*^)^ (*s_i_*) from this ccdf, and add it to the conditioning data set, now of dimension *n* + *i*.
4. Repeat step 3 until all *N* nodes along the random path are visited.

The probability of soil heavy metal at *s* exceeding the control standard (*z_c_*) can be denoted by *prob*[*z*(*s*) > *z_c_* ] [18,19]:

(11) $$prob[z(s)>z_{c}]=n(s)/1000$$

where *n*(*s*) is the number of realizations if *z*(*s*) is higher than the control standard in the 1000 SIS realizations.

## 3. Results and Discussion

### 3.1. Basic Statistics

Table 1 summarizes the descriptive statistics of the investigated four heavy metals (Cr, Cu, Ni, and Zn) from the original 1,082 samples. In Taiwan, the pollution control standards (maximum allowable concentrations) for the investigated heavy metals are as follows (in mg/kg): Cr 250, Cu 200, Ni: 200 and Zn 600. Table 1 lists 286 samples for Cr, 395 samples for Cu, 622 samples for Ni, and 336 samples for Zn over the control standards. Moreover, the high variability of the pollutant concentrations at various heavy metals requires a detailed evaluation and interpretation. The application of various methods is an efficient tool in achieving better understanding of the hazardous state of the soil pollution. It seems recommendable to combine various approaches instead of relying only on one of them to gain better information of the pollutions, such as the KDE, geostatistical methods (*i.e.*, the IK and SIS).

### 3.2. Point Pattern Analysis Using Kernel Density Estimation (KDE)

**Figure 2:**
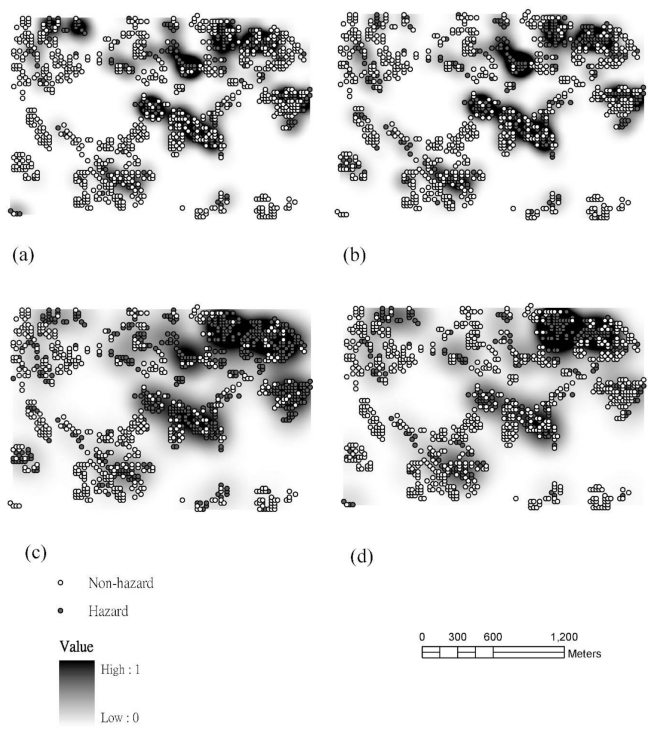
The kernel density maps (stretched to min–max range) of (a) Cr (b) Cu (c) Ni (d) Zn.

Figure 2 shows the hotspot patterns of soil pollutants on the kernel density map. The Kernel Density Estimation (KDE) transforms a dot pattern into a continuous surface, providing a more useful representation of soil pollution distributions, allowing for easier detection of possible pollution hotspots [24]. Results show that the hotspots associated with the four heavy metals in the study area are often multiple. We found that soil pollution hotspots were more clearly defined using KDE, probably because of the clustered distribution of soil pollution occurrences. The spatial patterns also reveal Cr hotspots near industrial plants and irrigation systems in the study area. The areas with Cu hotspots are in the central and eastern parts of the study area in the vicinity of the industrial plants and irrigation systems. Hotspots of Ni are particularly highly distributed throughout the northeast part of the studied area. The areas with Zn hotspots are close to the industrial plants and irrigation systems in the northwest. However, there are potentially contaminated sites that are hidden, especially in the areas where the history of land use is complicated and the sources of imported soils are usually unknown [27]. The maps show that the area with high susceptibility of pollution is along the industrial plants and the irrigation systems. The KDE results match the previous studies showing that the distributions of background heavy metals and pollution sources correlated with industrial plants and irrigation channels [15]. These industrial plants are suspected of discharging wastewater into irrigation systems in the study area [12,15,16,25].

### 3.3. Sampling Density and Spatial Interpolation of Probability Exceeding Control Standards Using the IK and SIS Approaches

The hazardous probability that heavy metal concentrations exceed control standards at any of the unsampled sites is determined by geostatistical methods (*i.e.*, IK and SIS). Moreover, the spatial distribution of hazardous probability can be characterized by an indicator variogram. The variance is estimated as a function of a variogram model, where the variogram is calculated using the relative locations of the samples. Table 2 lists the parameters of indicator variograms for the four heavy metals. In the indicator variograms, the fitted ranges, the nugget effects and the sills are 120–249 m, 0.0206–0.0251 and 0.187–0.235 (Table 2), respectively. The results show that the sill value for Ni is the largest one. Based on the cases, the higher sill corresponds to greater variability in the probability map. Spatial structure analysis has been identified to be a useful tool in illustrating the spatial patterns of variables, and a necessary basis for a number of other spatial analysis procedures, such as kriging analysis [28]. Furthermore, SIS realizations are performed based on the indicator variogram models for the 25th, 50th, and 75th percentiles of the sample distribution (Table 3) of original samples for Cr, Cu, Ni, and Zn in the study area.

**Figure 3:**
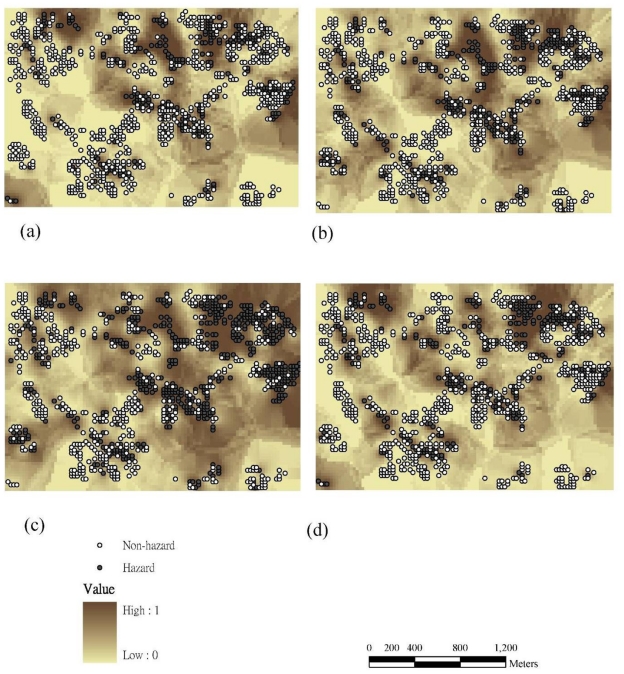
The probability maps of (a) Cr (b) Cu (c) Ni (d) Zn using indicator kriging based on 1,082 samples.

**Figure 4:**
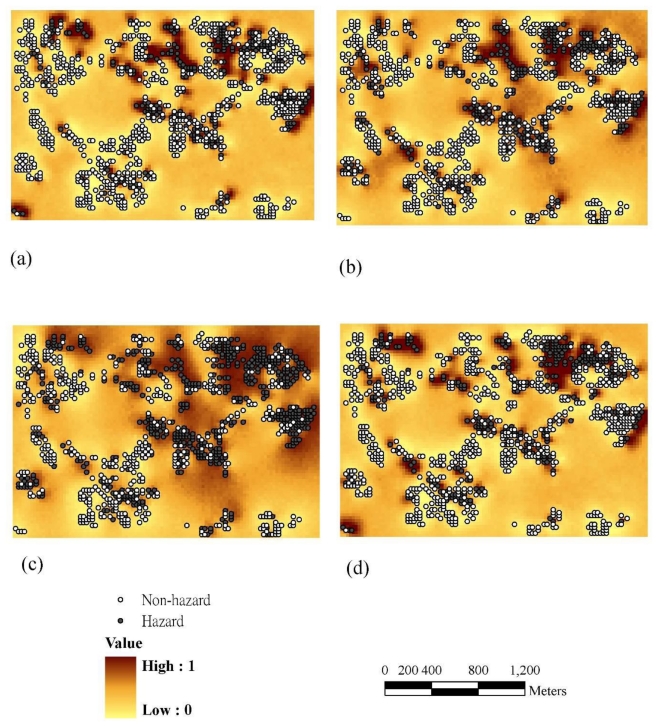
The probability maps of (a) Cr (b) Cu (c) Ni (d) Zn in 1000 realizations using sequential indicator simulation based on 1,082 samples.

Figures 3 and 4 show the probability maps for sites where Cr, Cu, Ni, and Zn exceed the control standards based on the IK and 1000 SIS realizations (by Equations 5 and 11).

The results demonstrate that the hotspots of hazard probability for Cr and Cu are similar. The spatial patterns of hazard probability also reveal hotspots of Cr near industrial plants and irrigation systems in the study area. The Cu hotspots are located in the central and east-northern parts of the study area in the vicinity of industrial plants and irrigation systems. The hotspots of Ni are distributed throughout the study area, except for the south-western part; and the areas with high concentrations of Zn are close to industrial plants and irrigation systems in the north-western part. Furthermore, all probability maps show that the multiple hotspots of hazard probability are close to industrial plants and irrigation systems in the study area.

Table 4 shows that polluted sampling density value is subjected to SIS probability exceeding regulatory thresholds with given critical probabilities (*p_c_* = 0.9, 0.8, 0.7 and 0.6) in the Cr, Cu, Ni, and Zn content of soil. Results show polluted sampling density increases as the critical probability increases. The polluted sampling density could be detected using KDE when delineating contaminations based on the original samples. In the study area, polluted sampling density range from 0.00023 to 0.00036 (L/m^2^) However, density values for heavy metal Ni are the lowest among the four heavy metals. For long-term pollution monitoring, the Ni pollution sampling points could be increased primarily. Based on these results, the KDE method is an effective approach to make sure of sampling density in delineating heavy metal pollutions for further monitoring.

### 3.4. Comparisons of Hotspot Visualizations by Various Methods

These techniques such as KDE, IK and SIS are commonly used in exploratory spatial analyses and pattern resolution for soil pollution visualization of heavy metals. All three visualization methods that we used to explore the soil pollution intensity patterns showed similar results (Figures 2–4) near factories and irrigation systems. These methods showed generally consistent results, but differences existed. KDE is an efficient means of detecting soil pollution hotspots based on point data. Results show the pollution hotspots are consistent in the other two approaches (*i.e.*, IK and SIS). The KDE results also show multiple hotspots in the study area and may under-emphasized areas with heavy metal pollution. However, the hotspots when determined based on KDE are more conservative than the ones estimated by IK and SIS. Results imply that the KDE multiple hot spots may be under-emphasized heavy metal pollution zones in the study area (Figure 2). Estimation results indicate that the hazard probability patterns estimated by the SIS are less fragmented than those estimated by IK (Figures 3 and 4). SIS takes into account not only the spatial variation of observed data at sampled locations but also the variation in estimations at unsampled locations which kriging estimation ignores those factors [18,25]. In addition, the simulation approach modifies the failure of IK to reproduce clusters of large concentrations above the tolerable maximum [18]. Simulation generates equally likely sets of values for a variable, which are consistent with available *in-situ* measurements. This usually implies that the simulated values have the same mean and variogram as the original data; they may also have to coincide with the original data at sampling points [29]. Furthermore, the local uncertainty information obtained by the IK is not sufficient to quantify the uncertainty at several locations simultaneously. Future work could assess multi-location uncertainties using SIS for the delineation of soil pollution. In addition, several investigators have published evidence of dynamics in environmental management [30–33]. In the future study, the temporal analysis of pollutant concentrations could be further explored.

## 4. Conclusions

This study utilizes KDE and geostatistical techniques with 1,082 samples to delineate hazardous zones and quantify the risk of multiple pollutants in a contaminated area. Various methodologies show generally consistent results, but differences exist. The results demonstrate that KDE is an alternative means of determining hazardous hotspots of soil pollutants only using hazardous point data in the preliminary investigation. The polluted sampling density could be detected by using KDE with SIS delineation. Moreover, the geostatistical models are approaches for identifying the risk of hazard delineation and are highly promising for use in evaluating the susceptibility of heavy metals without surveying soil concentrations over an entire study area. All proposed methods can be extended to show that soil pollution is closely related to pollution sources such as industrial factories and the irrigation system in the study area. According to the spatial maps, model assessment of soil pollution hotspots enables remediation planners to help identify hazardous pollution areas. Integrating KDE and geostatistical methods, the KDE method is an effective approach to determine sampling density when delineating heavy metal pollutions by geostatistical methods. The information of spatial sampling density and hotspot pattern could be useful for long-term monitoring and assessment.

## Figures and Tables

**Table 1:** Descriptive statistics of heavy metals for 1,082 samples.

	Min (mg/kg)	Median (mg/kg)	Max (mg/kg)	Average (mg/kg)	SD (mg/kg)	Control standards (mg/kg)	Number of observances over control standards
Cr	22.6	119.0	3,070.0	194.0	212.5	250	286
Cu	11.0	116.0	3,810.0	194.7	222.7	200	395
Ni	21.3	189.2	4,020.0	271.3	259.0	200	622
Zn	60.5	337.0	7,850.0	526.4	549.6	600	336

Min: minimum; Max: maximum; SD: standard deviation.

**Table 2:** Indicator variogram models for heavy metals.

	Threshold (mg/kg)	Model	C_0_	C_0_+C	R (m)	RSS	*r*^2^
Cr	250	Exp.	0.0237	0.1874	120	2.52E-04	0.859
Cu	200	Exp.	0.0251	0.2202	135	3.08E-04	0.904
	
Ni	200	Exp.	0.0206	0.2352	249	3.39E-03	0.723
Zn	600	Exp.	0.0221	0.2042	147	6.05E-04	0.808

Exp.: Exponential model; C_0_: Nugget; C_0_+C: Sill; R: Range; RSS: Residual Sum of Squares; *r*^2^: Coefficient of determination

**Table 3:** Indicator variogram models for the 25th, 50th, and 75th percentiles of heavy metals in 1,082 samples.

Heavy metal		Model	Parameters			RSS	*r*^2^
			C_0_	C_0_+C	R (m)		
Cr	25%	Exp.	0.020	0.184	216	1.730E-03	0.722
	50%	Exp.	0.026	0.247	171	1.202E-03	0.807
	75%	Exp.	0.025	0.190	120	2.075E-04	0.852
Cu	25%	Exp.	0.017	0.184	240	2.008E-03	0.737
	50%	Exp.	0.025	0.247	186	7.016E-04	0.899
	75%	Exp.	0.024	0.190	108	5.293E-04	0.663
Ni	25%	Exp.	0.015	0.179	222	2.614E-03	0.634
	50%	Exp.	0.022	0.237	228	3.608E-03	0.671
	75%	Exp.	0.018	0.183	159	5.723E-04	0.805
Zn	25%	Exp.	0.024	0.190	222	1.464E-03	0.768
	50%	Exp.	0.028	0.250	171	3.795E-04	0.936
	75%	Exp.	0.021	0.189	144	8.077E-03	0.710

Exp.: Exponential model; C_0_: Nugget; C_0_+C: Sill; R: Range; RSS: Residual Sum of Squares; *r*^2^ : Coefficient of determination.

**Table 4:** Polluted sampling density value based on SIS probability criteria.

	Critical probability(*p_c_*)	Number of grid which value is over *p_c_*	Density value (L/m^2^)	
			Mean	Range
Cr	0.6	591	0.00028	0.00066
	0.7	467	0.00031	0.00066
	0.8	373	0.00033	0.00066
	0.9	310	0.00034	0.00066

Cu	0.6	851	0.00029	0.00082
	0.7	643	0.00032	0.00081
	0.8	505	0.00034	0.00080
	0.9	403	0.00036	0.00080

Ni	0.6	2,157	0.00023	0.00071
	0.7	1,554	0.00027	0.00070
	0.8	1,099	0.00030	0.00067
	0.9	773	0.00032	0.00067

Zn	0.6	709	0.00028	0.00079
	0.7	560	0.00030	0.00079
	0.8	453	0.00032	0.00079
	0.9	379	0.00033	0.00079
